# Quality Control of the Dietary Supplements Containing Selected Fat-Soluble Vitamins D and K

**DOI:** 10.3390/nu15071650

**Published:** 2023-03-28

**Authors:** Małgorzata Starek, Paweł Gumułka, Monika Dąbrowska

**Affiliations:** 1 Department of Inorganic and Analytical Chemistry, Faculty of Pharmacy, Jagiellonian University Medical College, 9 Medyczna St, 30-688 Kraków, Poland; 2 Doctoral School of Medical and Health Sciences, Jagiellonian University Medical College, 16 Łazarza St, 31-530 Kraków, Poland

**Keywords:** vitamin D, vitamin K, dietary supplements, quality control, TLC-densitometry

## Abstract

Nowadays, the most important aspect related to the use of dietary supplements seems to be their quality. There are many reports indicating their insufficient quality primarily related to a much lower content of ingredients or even their absence. Currently, there is an increasing interest in supplementing the diet with various kinds of supplements, including those containing combinations of vitamins and minerals, among which preparations with vitamin D are very popular. This is probably due to the reduced production of this vitamin, depending on the amount of time spent in the sun and the use of UV-filters. Very often, preparations with cholecalciferol also contain vitamin K_2_, which is associated with their synergistic effect. Therefore, the question arises about the effectiveness of supplementation, which may be correlated with the quality of commonly available dietary supplements. In the presented work, it was undertaken to develop optimal conditions for the qualitative and quantitative determination of vitamins D_2_, D_3_ and K_2_ in dietary supplements available in various forms, using thin-layer chromatography with densitometric detection. As a result, the methodology for analyzing the content of three vitamins from various matrices was developed, optimized and validated in accordance with ICH requirements. The obtained results allow us to conclude that it is reliable and meets the requirements for analytical procedures used in the analysis of medicinal products. Based on the results obtained for examined dietary supplements, it can be stated that the amount of vitamin D_3_ in analyzed products is basically similar to that declared by the manufacturer, in contrast to vitamin K_2_, the content of which is diverse. The developed methodology seems to be a good, low-cost and quick way to control the quality of dietary supplements so that they can supplement the human diet and be a wholesome product.

## 1. Introduction

Vitamins are a large group of organic substances involved in all basic functions of the body, enabling it to function properly. The body obtains vitamins mainly from food or supplied supplements. B, C and PP vitamins are soluble in water and absorbed by the body in necessary amounts, and the excess is excreted in the urine. In turn, fat-soluble vitamins include: vitamin A, D, E and K [1]. In terms of structure, they are derivatives of isoprene (more precisely isopentenol); they are polar, hydrophobic molecules. Due to their lipophilic nature, they can accumulate in the body, most often in the liver and adipose tissue, which can cause their toxicity in the state of excessive supply.

**Figure 1:**
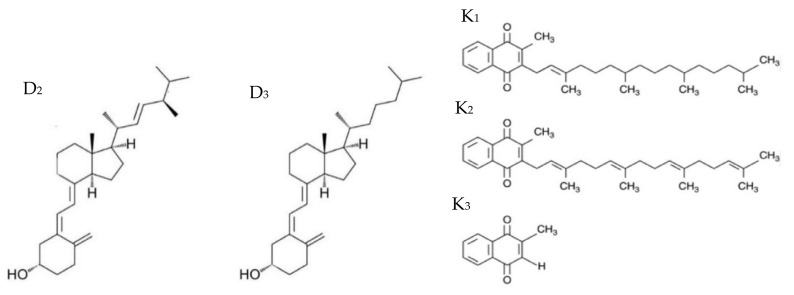
Structural formulas of vitamins D_2_, D_3_, K_1_, K_2_ and K_3_ [2,7].

Vitamin D, as a group of fat-soluble steroid compounds, was discovered in 1922 by Dr. McCollum [2,3], and includes vitamin D_3_ (cholecalciferol, calciol) synthesized in the skin of animals, vitamin D_2_ (ergocalciferol, ercalciol) found in plants and vitamin D_1_ found in cod liver oil. Both vitamin D_3_ and D_2_ occur in the human body, are synthesized under the influence of UV radiation (D_3_) or supplied with food (D_2_ and D_3_). Looking at the chemical structure of these compounds, it can be concluded that the difference lies only in the structure of the side chain. Vitamin D_2_ additionally has a methyl group (C22) and a double bond (C23-C24) (Figure 1) [4]. Cholecalciferol is formed from a cholesterol derivative, 7-dehydrocholesterol (provitamin D_3_), in the outer part of the human skin under the influence of UVB radiation (290–315 nm). A similar transformation path applies to the D_2_ form, with the difference that the starting compound is ergosterol, and the process takes place in plants and fungi [5,6].

Both forms of the vitamin must be converted to the active metabolite. This is done by double hydroxylation [5]. The entire process of vitamin D activation is regulated by negative feedback, based on the inhibition of hydroxylase activity by the negative effect of the active vitamin formed in excess on the parathyroid hormone [3]. 

The physiological functions of the vitamin D active form are usually related to the effects on the level of calcium and phosphate in the blood, through actions at the level of the small intestine, bones and kidneys [8]. On the basis of research dealing with the action of vitamin D at the molecular level, its interference in the proliferation and differentiation of cells, mainly in the skin, intestine and bone marrow, has been proven. By affecting the calcium and phosphate metabolism, vitamin D ensures proper bone mineralization and muscle function. The effect of deficiency of this vitamin is osteoporosis, muscle weakness, osteomalacia, and in children, rickets, growth disorders and skeletal deformation. It also affects the respiratory system, for a better response of the body to drugs used in the treatment of asthma. In a randomized study, Urashima et al. demonstrated that vitamin D deficiency increases the incidence of influenza A among school-aged children [9]. One should also remember about the role of calcipotriol in preventing inflammation of the colon, reducing the risk of developing diabetes, positive effect on the cardiovascular system and even possible participation in the course of carcinogenesis [4,10].

Vitamin K, discovered in 1930, comes in several forms (K_1_, K_2_, K_3_), two of which occur naturally (Figure 1). Chemically, they are derivatives of 2-methyl-1,4-naphthoquinone, differing in the length of the isoprenoid side chain in the C3 position and the number of unsaturated bonds in this chain. Vitamin K_1_ (phylloquinone; PK) is found in plants, while K_3_ (menadione) is a synthetic derivative without a side chain, characterized by the highest biological activity. The most important form for humans is the K_2_ form (menaquinone; MK-n, where n is the amount of unsaturated isoprenoid residues). The most important types are MK-4 and MK-7, which have the longest half-life in the body. Vitamin K_2_ is produced by saprophytic bacteria in the intestine from vitamin K_1_; it can also be absorbed with animal food or with the increasingly popular Japanese fermented soybean. Physiochemically, this group of compounds is water-insoluble, with the exception of vitamin K_3_, which in the form of bisulfite is water-soluble. All forms are stable in an acidic environment but may change properties when exposed to light [11]. All the functions of vitamin K in the body result from its participation in the modification of proteins, consisting in the gamma-carboxylation of glutamic acid in amino acids.

It can be seen that both vitamins—D and K—have a synergistic effect on many levels, e.g., by affecting the skeletal system. Menaquinone performs its function in maintaining the proper functioning of this system by increasing bone mineralization, which results from increased gamma-carboxylation of osteocalcin, and reduces the activity of osteoclasts. The effect of vitamin K on the occurrence of dementia syndromes was also observed, examining the correlation between the level of phylloquinone and apolipoprotein E, a factor in the development of Alzheimer’s disease. Participation in the prevention of calcification, i.e., the deposition of calcium and phosphate deposits in blood vessels, is not without significance. Scientific reports also indicate that vitamin K induces oxidative stress, which negatively affects cancer cells and directly affects their apoptosis [11,12,13].

Due to the many important functions performed in the human body by vitamins D and K, their possible deficiencies should be adequately supplemented. In general, more people are deficient in vitamin D than K, due to insufficient exposure to sunlight due to geographic location, but also the use of sunscreen and increasing avoidance of the sun for fear of the negative effects of radiation [14]. Separate cases are people who suffer from kidney or liver failure, which leads to vitamin D metabolism disorders and does not allow the production of the active form of the compound. Therefore, the guidelines are being updated, in which the team of experts defines the norms of the amount of vitamin D that should be followed during supplementation in the general population and in risk groups [8,15,16,17,18]. To determine the body’s supply of vitamin D, the level of 25-hydroxycholecalciferol [25(OH)D] in the blood should be determined, the optimal level of which is 75–200 nmol/L (30–80 ng/mL) [6,15]. Supplements used to supplement vitamin D deficiencies may contain its various forms and doses. The most common ingredient is cholecalciferol (D_3_), while ergocalciferol (D_2_) is also available on the American market [3]. Available products also contain vitamin D_3_ in combination with other vitamins (including K) and calcium. Vitamin D_3_ analogs and metabolites are not routinely used in the supplementation of healthy people; calcidiol is sometimes used in the case of liver failure, while calcitriol and α-calcidol in people with kidney failure [15].

We talk about vitamin K deficiency much less frequently. The most vulnerable to them are newborns, people with malabsorption syndrome and liver disorders and those treated with anticoagulants [19]. In the case of identified deficiencies, supplementation is recommended. The daily dose of all forms of vitamin K should be 50–120 μg/day, however, it refers to the demand related to the synthesis of coagulation factors in the liver. Unfortunately, we do not have the vitamin K values necessary for other tissues and processes [11].

Extraction of substances from materials, i.e., dietary supplements, food or blood, is a key stage in the quantification of substances, affecting the accuracy and reliability of the obtained analysis result. The relatively small amount of vitamins in these materials and the possibility of degradation in the conditions of the extraction process seem to be a problem. To prevent thermal degradation, the process can be carried out at room temperature, and the stability can be increased by the addition of appropriate antioxidants [4,20,21,22,23]. In products of natural origin, vitamin D can be present in free form or as esters. The extraction process can be started with saponification of the analyzed sample, thanks to which the vitamin in the form of esters will be released, but hot saponification involves the risk of thermal isomerization of the vitamin. Hot or cold extraction can also be carried out directly using organic solvents, mainly dichloromethane, hexane, methanol, diethyl ether, chloroform and their mixtures [24,25,26].

High-performance liquid chromatography (HPLC) has become a routine method for the analysis of vitamin D and its metabolites. Studies of the content of vitamin D_2_ and D_3_ in medicinal preparations were carried out using a C18 column, a mobile phase containing acetonitrile and methanol and a diode array detector (DAD) [27]. A similar method was used for the determination of vitamin D_3_ in pharmaceutical preparations, and additionally, the stability of the vitamin and the analysis of degradation products were determined [28]. Vitamins D_2_ and D_3_ were quantified in milk using HPLC with methanol as the mobile phase, the optimum process temperature was 15 °C [29]. Simultaneous determination of various fat-soluble vitamins and provitamins (i.e., A, D_2_, D_3_, E, K_1_) was established [30]. Vitamin D metabolites (25(OH)D_3_ in addition to 25(OH)D_2_) in serum were also determined using UV detection, a LiChrospher 60 RP column and a mobile phase composed of methanol and water [31]. HPLC with fluorescence detection and chemiluminescent reaction preceded by UV irradiation were used to analyze vitamin K in various matrices [32]. In multivitamin preparations, phylloquinone was determined by HPLC at 248 nm using 95% methanol as the mobile phase [33]. The RP-HPLC technique was also used to analyze menaquinone and its derivatives in microbiological material [34], in animal feed, using an amperometric detector [35] and together with phylloquinone in human milk [36]. The RP-HPLC-MS technique was used to analyze the content of vitamins D and K in functional food [37]. Using liquid chromatography coupled with tandem mass spectrometry (HPLC-MS/MS), vitamin D metabolites in the blood were determined [38]. In combination with isotope dilution analysis, the content of 25(OH)D_2_ and 25(OH)D_3_ was assessed [39]. A procedure for the quantitative determination of cholecalciferol and ergocalciferol in serum has also been developed [40]. Using LC-MS/MS determinations of phylloquinone and menaquinone in serum [41], phylloquinone, menaquinone-4 (MK-4) and phylloquinone epoxide in blood and serum [42], and eleven vitamins, including vitamin K, in various materials, such as food, blood and feces [43], were carried out. The high selectivity of the LC-MS/MS technique is used to determine 25-OH-cholecalciferol and 25-OH-ergocalciferol in the blood, or the concentration of the inactive metabolite, 3-epi-OH-vitamin D_3_, which is mainly found in infants [44]. Vitamin D was also determined by thin-layer chromatography (TLC) in various samples in the form of metabolites, single compound and in combination with other vitamins and lipids. The difficulty was the fact that it can be easily degraded under the influence of environmental factors, e.g., oxygen [45,46]. Visualization of spots on the chromatogram was made in UV light and after spraying the plate with various reagents [47]. Janecke and Maass-Goebels extracted vitamin D from mixtures with other sterols and their degradation products, and then used TLC with different variants of mobile phases [48]. Vitamins A, D_2_ and E were determined using RP-18 plates and the mobile phase: acetonitrile—benzene—chloroform [45]. Vitamin D_2_ in tablets was also determined on silica gel 60F_254_ using mixtures of hexane and ether or cyclohexane and ethyl acetate as the mobile phase [49,50]. High-performance thin-layer chromatography (HPTLC) was used to study the content of cholecalciferol in fish liver oil [45,51], for the separation of vitamin D_3_ in the presence of its metabolites [52], and determination of 1,25-dihydroxyvitamin D_3_ along with other plasma metabolites [53]. The TLC technique was also used to analyze mixtures containing vitamins D_2_, D_3_, K_1_, K_2_ (MK-4), K_3_ and A [54] and D_3_ and K_1_ in preparations acting as rodenticides [55].

The presence of vitamin K_1_ and MK-4 in the liver of rats was tested using an eluent containing petroleum ether and diethyl ether [56], and in the reverse phase system, in bovine liver [57]. The chromatographic-spectrophotometric methods for the determination of vitamins K_1_, K_3_ and K_4_ in drugs (tablets, dragees and injections) [58], and K_2_, K_3_ and K_4_ in tablets and solutions [59,60,61] were carried out. Simultaneous quantification of vitamin K analogues in drugs (tablets and injectable solutions) and food products, fruits and vegetables was also performed [62].

For the analysis of vitamins D and K by gas chromatography (GC), derivatization of the sample is required. With regard to vitamin D, the analysis after trimethylsilylation turned out to be the best solution [4]. Menadione in dietary supplements was analyzed using the GC with a flame ionization detector (FID) and in animal feed using the combined GC-FT-IR technique [21]. GC coupled with mass spectrometry (GC-MS) was used to separate cis/trans isomers of vitamin K [22].

The micelle electrokinetic chromatography (MEKC) technique was used to analyze vitamin D_2_ multivitamin tablets [63,64]. Simultaneous determination of lipophilic and lipophobic vitamins was also carried out using the capillary electrochromatography technique (CEC) [65]. Whereas, menaquinone in bacterial cells was determined using specific acid binding agents, followed by analysis using ion exchange chromatography and electrophoresis [36].

Spectrometric methods are based on the reaction of vitamin K_3_ with various substrates, resulting in products that are colored or show absorption of UV radiation [66,67]. The method of condensation of vitamin K_3_ with acetylacetone and diethylmalonate in ammonia medium [68], determination of K_2_ after reduction with potassium borohydride and phylloquinone after reaction with xanthine hydride or diethylthiocarbamate [69] were also described. Vitamin D_3_ in capsules and tablets was determined spectrometrically at 264 nm [70], as well as after the complexation with molecular iodine [71]. The analysis of vitamins D_2_ and K_1_ was carried out in the presence of rutin, however, due to similar absorption maxima, it was necessary to separate these vitamins by TLC beforehand [72].

Techniques often used to determine vitamin D and its metabolites are immunochemical methods, although they are mainly used to measure the concentration of metabolites in the blood to determine possible deficiencies. The problem with these assays is, first, that the antibody has different affinities for the vitamin D_2_ and D_3_ metabolites, and secondly, poor accuracy. A modification of this method is the radioimmunoassay (RIA) using radioactive labeling ligands, most often ^125^I [31,40,73]. The chemiluminescent assay method (CLIA) is becoming more and more popular, which uses a UV-emitting tag to facilitate detection, or enzymes that catalyze reactions (ELISA method) [74,75,76].

For the determination of vitamin D, electrochemical analysis was also used using the technique of labeling the analyzed compound, e.g., with 4-ferrocenylmethyl-1,2,4-triazolin-3,5-dione, which reacted with 25(OH)D [77] or enzyme CYP 27B1, which was placed on the electrode and then measured by voltammetry [78]. While, phylloquinone was analyzed using cyclic voltammetry, using a flat electrode with carbon glass, and menadione in medicinal preparations was tested potentiometrically, after oxidation with sulfite [36].

As is well known, vitamins are present in various food products in a different way in terms of type and amount. Only a balanced diet and a proper lifestyle can cover the demand for the necessary amounts of these ingredients [79]. The number of commonly available products containing vitamins is huge, and their consumption is very widespread in all age groups. According to the Food and Nutrition Safety Act, a dietary supplement is a means intended to supplement a proper diet, constituting a source of minerals, vitamins and substances with a nutritional or physiological effect [80]. The form in which they are sold, e.g., capsules, tablets, and the fact that the main place of their sale is a pharmacy, contribute to attributing them medicinal properties. In fact, the requirements for medicinal products and dietary supplements, as well as the marketing authorization procedure for both, are significantly different. Dietary supplements are classified as foodstuffs, and it should not be used as a substitute for medicinals [81]. The requirement for a dietary supplement to be placed on the market is the manufacturer’s notification to the Chief Sanitary Inspector and the presentation of a draft product label. It should be remembered that, although they often contain the same active substance as drugs, they do not have to possess a full description of studies demonstrating their quality and effectiveness. The stability of the compound contained in the supplement is also not controlled, and side effects related to their intake are not monitored [82]. Hence, the need for constant control of their quality, especially in the case of fat-soluble vitamins, overdose of which can cause serious poisoning [83]. There are also more and more reports indicating their insufficient quality associated primarily with a much lower content of ingredients or even their absence [84]. Differences can be found both in different products containing a given ingredient and in different series of the same supplement from one manufacturer [85]. Studies conducted in Italy have shown three orders of magnitude higher vitamin D content than the value declared on the packaging. The same supplement obtained from a different source contained a lower than expected amount of the active substance [24]. That is why a thorough analysis of these products seems so important in order to make more and more people aware of the need to take care of their quality, so that they can actually serve as a supplementary element of the human diet and be a wholesome product.

In the scientific literature presenting analytical methods, including chromatographic ones, changes related to the need to introduce new practices leading to environmental protection (‘green chemistry’) have been observed for some time [86]. New solutions are emerging that lead to reductions in cost, time, complexity of analytical procedures, energy, solvents and waste production. TLC is a commonly used analytical technique that gives a quick answer to the question of how many components are in the mixture, and in combination with densitometric detection, also informs about the amount of components in the sample. It is one of the easiest and most versatile methods due to its low cost, simplicity, fast development time, high sensitivity and good reproducibility. Scientists routinely use TLC in many industries and research fields, e.g., to identify mixture ingredients, check purity, compound stability or monitor reaction kinetics [87,88].

The number of dietary supplements entering the market is growing rapidly each year, and the large number of new products limits the possibility of constant control of their quality. At the same time, the recommendations of vitamin D supplementation in various age groups contributed to the increase in the number of dietary supplements containing it. The main objective of the study was to determine the content of vitamins D_3_ and K_2_ (MK-4 form) in dietary supplements available on the Polish market. It was decided to (i) optimize the conditions for the separation of vitamins D_3_, D_2_ and K_2_ using the TLC technique with densitometric detection, (ii) develop a method of extracting vitamins from various forms of dietary supplements, (iii) validate the developed methodology, and (iv) use the developed procedure for qualitative and quantitative analysis of vitamins D and K_2_ in dietary supplements. For the analysis of supplements, a procedure analogous to that used in the analysis of drugs will be used.

## 2. Materials and Methods

### 2.1. Apparatus and Equipment

Applicator Linomat V (Camag, Muttenz, Switzerland) with a 100-microL syringe (Hamilton Bonaduz, Switzerland); UV Cabinet 3 Lamp (Camag, Muttenz, Switzerland); Chromatographic chambers (Sigma Aldrich, Saint Louis, MO, USA); Densitometer TLC Acanner 3 (Camag, Muttenz, Switzerland); Chromatographic plates (Merck, Darmstadt, Germany): TLC Silicagel 60F_254_ no 1.05554, DC Silicagel 60 RP-18 F_254S_ no 1.05559, DC Alufolien Cellulose F no 1.05574, DC Alufolien Kieselgur F_254_ no 5568, DC Alufolien Polyamid 11 F_254_ no 5555, DC Alufolien Aluminiunoxid 60 F_254_ neutral Typ E no 5550.

### 2.2. Reagents

All used reagents: *n*-hexane, ethyl acetate, chloroform, toluene (POCH, Gliwice, Poland), acetone, cyclohexane, glacial acetic acid (Chempur, Piekary Śląskie, Poland), methanol, acetonitryl (Merck, Darmstadt, Germany) were analytical grade. β-cyclodextrin and liquid paraffin were purchased from Sigma Aldrich (Poznań, Poland) and COEL (Kraków, Poland).

### 2.3. Standard Substances

Standard substances of analyzed vitamins, Vitamin D_3_ (C9756), Vitamin D_2_ (PHR1238) and Vitamin K_2_ (MK-4; V9378) were purchased from Sigma Aldrich (Poznań, Poland).

### 2.4. Dietary Supplements Used for Analysis

All preparations and supplements from various manufacturers were purchased in local pharmacies. Some of them exceeded the expiry date within the time of the study. The analyzed 25 dietary supplements containing vitamin D_3_ and/or K_2_ were in various forms, such as tablets, power, capsules, drops, with declared contents of 5–500 µg per 1 tablet, 1 dose, 1 capsule or 1 mL. In the present research, the complements were coded with letters from A to Z, for reasons of confidentiality.

### 2.5. Optimization of Analysis Conditions

The selection of optimal conditions for the simultaneous determination of vitamins D_2_, D_3_ and K_2_ by TLC was started based on the available publications. For this purpose, methanol standard solutions of vitamins with concentrations of about 0.4 μg/μL were prepared in measuring flasks, which were then applied to chromatographic plates (TLC Silicagel 60F_254_, DC-Silicagel 60 RP-18 F_254S_, DC-Alufolien Cellulose F, DC-Alufolien Kieselgur F_254_, DC-Alufolien Polyamid 11 F_254_, DC-Alufolien Aluminiunoxid 60 F_254_ neutral Typ E) in the amount of 5, 10 and 20 μL per spot (single and their mixture). The plates were developed at different distances (10 to 20 cm) using mobile phases with varying amounts of different solvents. Due to the unsatisfactory effects, it was decided to modify the conditions by activating the plates (chloroform, methanol, 1% (*w*/*v*) β-cyclodextrin aqueous solution, 5, 10 and 15% (*v*/*v*) paraffin solution in cyclohexane, heating in an incubator at 60 °C for 2 and 24 h). The process of activation with a paraffin solution was carried out both by unrolling the plates in a chromatographic chamber and by immersion directly in the paraffin solution. The plates were then dried in an incubator (5, 10, 30 min) at an elevated temperature (30, 60, 80 °C). Standard solutions of vitamins were also modified. For this purpose, a sample of each of the standard substances was dissolved in 3 mL of methanol and made up to 5 mL with a 1% (*w*/*v*) β-cyclodextrin aqueous solution. The description of the conditions and the results of the carried-out tests are collected in Table S1.

To sum up, after applying many different modifications of the analysis conditions for vitamins D_2_, D_3_ and K_2_ next to each other, the most optimal separation was observed using TLC Silicagel 60F_254_ plates activated with a solution of 10% (*v*/*v*) paraffin in cyclohexane, then dried for 10 min at 60 °C, developed in a mixture of methanol-water (19:1, *v*/*v*) as the mobile phase, over a distance of 12 cm. The above analysis conditions allowed to obtain the following retardation factor (R_F_) values for individual vitamins: for K_2_—0.18, D_3_—0.53 and D_2_—0.58.

### 2.6. Stability Testing

The next stage of the work consisted in checking the stability of solutions containing vitamin D and K_2_ under the influence of solar radiation and temperature. For this purpose, methanolic solutions were left for 48 h in a sunny place. Similarly prepared solutions were subjected to an elevated temperature (in tightly closed dark glass vials), heating them for 24 h at 60 °C and for 2 h at 90 °C. After the specified time, the samples were applied and analyzed under the conditions described above. At the same time, a test was carried out with samples of identical composition but left in a shaded place and stored at room temperature for the same period of time, as controls.

### 2.7. Validation of the Method

Validation in relation to an analytical method is a process leading to documented confirmation of the suitability of a given method for a specific application and experimental proof of its reliability [89]. Not all validation parameters need to be estimated for every analytical process. Validation should cover primarily those features that determine the suitability of the method for the assumed task. An assay of substances contained in drugs requires ensuring that the used procedure meets the criteria of linearity, precision and accuracy and LOD and LOQ values.

Linearity is a parameter that defines the directly proportional relationship between the measured values (MW) and the concentration of the analyte (c). In order to verify the compliance of the empirical properties of the tested model with the theoretical requirements (checking the robustness of the regression model), a residual analysis was also performed and the values of the Cook and Mahalanobis distances were determined. The limit of detection (LOD = 3.3∙S_b_/a; S_b_ is the standard deviation of the response and a is the slope of the regression curve) is the smallest amount of a test compound that can be detected by a given method, but not necessarily quantified. The limit of quantification (LOQ = 10∙S_b_/a) is the smallest amount of a test compound that can be quantified with optimal accuracy and precision using a given analytical method. The precision of a method includes two parameters: repeatability (the same conditions, in short intervals of time) and reproducibility (different conditions, i.e., different laboratory, apparatus, analyst). The standard deviation and relative standard deviation should be designated. Accuracy, parameter that determines the compliance of the obtained results with real values can be determined by percentage of recovery, according to the formula: %A = (x/y) ∙ 100%, where x—the determined amount of the test substance in the sample, y—the actual amount of the test substance in the sample. This test is based on adding known amounts of the test substance (80, 100 and 120%) to the sample, and then determining the total amount by a given analytical method. It is assumed that the percentage of recovery should be in the range of 95–105%. The sensitivity of the method is defined as a change in the value of the measured signal under the influence of a change in the amount of the analyte. It is presented as the slope of the calibration curve. Selectivity is the ability of a method to determine a single compound in a composite sample. On the other hand, it means the ability of a method to unequivocally confirm the presence of a given substance in a sample.

### 2.8. Analysis of the Vitamins Content in Chosen Dietary Supplements

In the next stage of the work, it was decided to examine the content of vitamins D and K_2_ in dietary supplements available on the Polish market. The study concerned the analysis of the content of vitamins D_3_ and K_2_ in selected dietary supplements in the form of tablets, powder, capsules and drops. The analysis began with attempts to optimize the efficiency of the extraction process.

After crushing a few tablets in a mortar, the tablet mass was weighed in an amount corresponding to the concentration of the standard solution of the appropriate vitamin (approx. 0.4 μg/μL). Methanol was then added to the weighed sample, the samples were shaken vigorously for 10, 20 and 30 min and then placed in a rocker shaker for 15, 30, 45 and 60 min. After shaking, the solutions were filtered. Similarly, a series of solutions were prepared, which, after shaking, were left in contact with the precipitate for 24 h in a refrigerator (temp. approx. 5 °C). At the same time, solutions of supplements were prepared, to which a certain amount of reference substances was added and extracted as described above. After filtering the solutions, they were applied to the prepared plates in an amount corresponding to the content of 1 μg of vitamin K_2_ and 3 μg of vitamin D_2_ and D_3_, and then subjected to chromatographic analysis in accordance with the previously developed procedure. In addition, standard solutions of vitamins D_2_, D_3_ and K_2_ were applied to each plate. Based on the obtained results, it was decided that the extraction of the components of the supplement solutions occurred with the highest efficiency after 30 min of vigorous shaking and 30 min of slow rocking stirring. Leaving the solution in contact with the solvent for 24 h before filtration did not bring significant differences.

The efficiency of vitamin extraction from preparations in the form of drops and soft capsules was checked after preparing solutions of selected preparations (Product Q and S) in various solvents. The obtained discrepancies in the amount of the component released from the preparation were an introduction to modifying this process by intensive mixing for 5, 15 and 30 min, and then using a rocking shaker for 1, 3 and 5 h. Equal amounts (in terms of the amount of vitamin D_3_) of the mixtures and the standard solution were analyzed under fixed conditions. The largest amount of vitamin was extracted using acetone, *n*-hexane and methanol. In the case of acetone solutions, in addition to the vitamin D_3_ spot, additional large spots were observed, probably due to the presence of other components of the preparation. *n*-Hexane mixes with the oil content of the supplement to form a homogeneous mixture, which results in tailing of the peaks. In contrast, methanol is immiscible with oil, so the vitamin can be extracted into the organic solvent layer, while the fatty acids remain in the oil layer. The obtained results suggest that the best extraction efficiency was obtained after 15-min intensive shaking of the solution of the preparation with methanol, followed by a 3-h mixing stage on a rocker shaker. After preparation, the solutions were subjected to chromatographic-densitometric analysis by applying dietary supplement solutions in a volume of 10 or 20 μL of solutions, which corresponded to the amount of 2 μg of vitamin D per spot (calculated according to the content declared by the manufacturer). At the same time, methanolic vitamin D_3_ standard solution in the amount of 10 μL was applied to each plate.

## 3. Results and Discussion

Given the vast amount of dietary supplements available around the world, quality control measures appear to be insufficient and label claims may mislead consumers. For this reason, one of the objectives of our work was to check whether products containing vitamins D and K meet the quality requirements. Then, we assessed their quality in accordance with the procedure used for drugs. For this purpose, a new analytical procedure was developed that allows the simultaneous qualitative and quantitative determination of the content of vitamins D_2_, D_3_ and K_2_ side by side.

**Figure 2:**
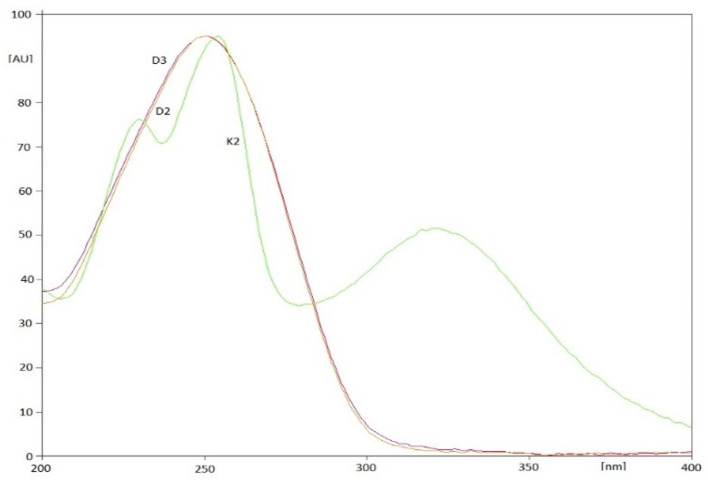
Absorption spectra in the UV range obtained for the standard solutions of the tested vitamins D_2_, D_3_ and K_2_.

By optimizing the conditions for the separation and determination of vitamins, it was found that the most optimal separation occurs with the use of TLC Silicagel 60F_254_ plates activated by direct immersion in a 10% (*v*/*v*) solution of paraffin in cyclohexane for 5 min, followed by drying in an incubator at 60 °C for 10 min. Plates prepared in this way were developed in a chromatographic chamber saturated with the mobile phase (for 15 min) along a distance of 12 cm, using a mixture of methanol-water (19:1, *v*/*v*). The total development time was approx. 2.5 h. The air-dried plates were scanned densitometrically at an analytical wavelength of 254 nm. The choice of wavelength was made on the basis of absorption spectra for standard solutions of vitamins D_2_, D_3_ and K_2_ recorded directly from chromatograms (Figure 2). On the basis of the obtained R_F_ values and absorption spectra in the range of 200–400 nm, registered for reference substances and ingredients of supplements, individual vitamins were identified. However, for quantitative determinations, peak area values recorded on densitograms were used.

At the same time, the described attempts to optimize the analysis conditions made it possible to determine the stability of the tested vitamin solutions. On the densitograms obtained for the solutions of the tested vitamins exposed to solar radiation and temperature, no additional peaks were observed that could come from the decomposition products of vitamin D. However, vitamin K_2_ turned out to be less stable in solutions. For this reason, all used standard and sample solutions were prepared immediately before use and possibly stored overnight at a reduced temperature (+4 °C).

**Figure 3:**
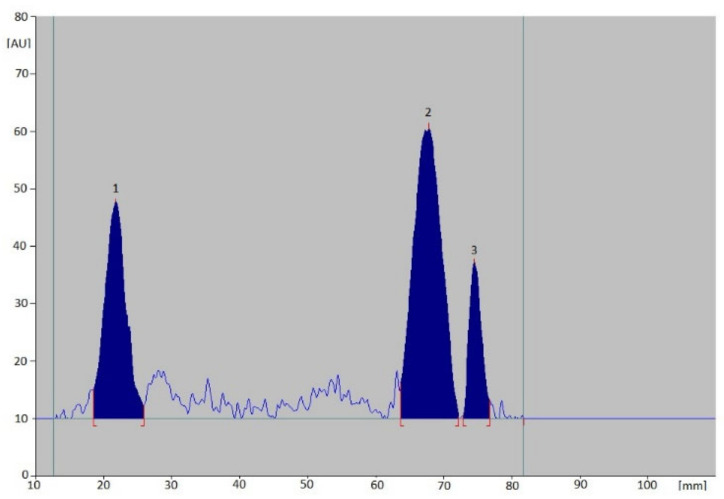
An example densitogram obtained for the standard solutions of the analyzed vitamins (1—K_2_, 2—D_3_, 3—D_2_).

The developed conditions allowed to obtain dense spots on the chromatograms and well-developed peaks on the densitograms. The analysis of the recorded densitograms allowed to determine the values of R_F_ coefficients for individual vitamins, which are: 0.18 for K_2_, 0.53 for D_3_ and 0.58 for D_2_ (Figure 3).

Based on the distances of individual peaks in the densitograms, the values of the separation coefficient (R_s_) were calculated, determining the extent to which two adjacent spots on the chromatogram were separated. If R_s_ is greater than 1, it proves complete separation of the substance, the greater the positive value of the parameter. The separation coefficients were calculated according to the following formula: R_s_ = 2∙(Z_2_ − Z_1_)/(W_1_ + W_2_), where: Z_1_, Z_2_—the distance of the maxima of the adjacent peaks in the densitogram from the starting line (distances of the centers of the spots from the starting line), W_1_, W_2_—peak widths in the densitogram (spot widths measured along the development path).

The obtained R_s_ values confirm previous observations and a good separation of vitamin K_2_ from D_2_ and D_3_, but insufficient separation of vitamins D_2_ and D_3_ to be able to simultaneously determine them (especially with a significantly higher concentration of one of these compounds): R_s_ (D_2_/D_3_) = 1.13, R_s_ (D_2_/K_2_) = 5.94, R_s_ (D_3_/K_2_) = 9.72. The above separation conditions make it possible to confirm the identity of all three vitamins next to each other in the mixture, at tested concentrations of vitamins D_2_ and D_3_ levels.

**Figure 4:**
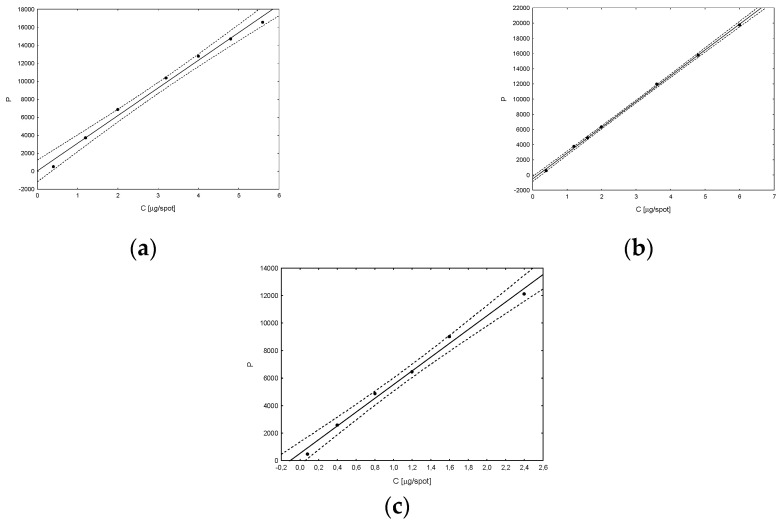
Calibration curves (peak area vs concentration; ±0.95% conf. int.) for vitamin standard solutions: D_2_ (**a**), D_3_ (**b**) and K_2_ (**c**).

**Figure 5:**
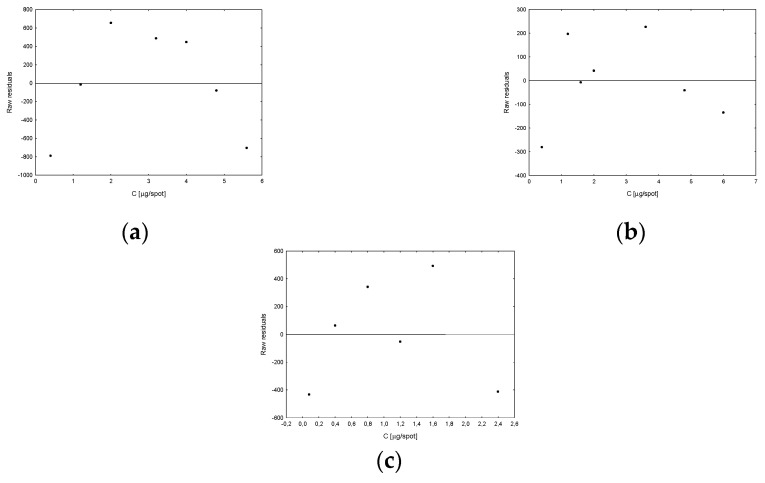
Plots of residuals calculated for vitamin D_2_ (**a**), D_3_ (**b**) and K_2_ (**c**).

Next, the developed procedure was validated in order to confirm its reliability. In order to determine linearity, standard solutions of vitamins were prepared with concentrations: for vitamin D_2_ 0.40–5.60 μg/spot, for D_3_ 0.40–6.00 μg/spot and for K_2_ 0.08–2.40 μg/spot. Then, they were applied to chromatographic plates and subjected to further analysis according to the procedure described above. The obtained values of peak areas corresponding to individual vitamins were the basis for plotting calibration curves (Figure 4). The plots of residuals show how much the obtained residuals deviate from the regression line and the location of the points looks random (Figure 5). After statistical analysis of the obtained results, the values of individual regression parameters were determined, which are summarized in Table 1.

On the basis of the regression data, the values of LOD and LOQ parameters for individual vitamins were determined using the formulas provided earlier. The results obtained are presented in Table 1. Graphical analysis of the residuals allows to determine the consistency of the distribution of residuals with the normal distribution. The values of the correlation coefficients of the residual dependence on the concentration reach very low values (about 10^−6^), which confirms the practically random location of the measurement points and the fulfillment of the assumptions of the adopted regression model. Cook distances, which measure the extent to which the regression coefficients change when the indicated case is removed, take values of the same order and no greater than 1, indicating that no point had a significant effect on the bias of the regression coefficients. However, Mahalanobis distances (i.e., the distance of a given measurement point from the center) confirm that no observations are outliers. Thus, it can be confirmed that the determined values of the regression model are correct (Table 1).

In order to determine the precision, methanolic standard solutions of vitamins D_2_ and D_3_ with concentrations of 0.40 μg/μL and vitamin K_2_ standard solution with a concentration of 0.04 μg/μL were used. They were applied several times on the plates in the volume of 3 μL of vitamin D_2_ and D_3_ solutions and 10 μL of vitamin K_2_ and analyzed under the conditions described above. Intermediate precision determinations were carried out in a similar way. Statistical analysis was performed on the basis of the recorded peak areas, the results of which are presented in Table 1. The accuracy of the developed method was assessed by analyzing the recovery of a known amount of standard substances of vitamins D_3_ and K_2_ added (in the amount of 80, 100 and 120%, respectively) to one of the dietary supplements containing both vitamins. The prepared solutions were subjected to chromatographic analysis under the conditions described earlier. At the same time, a solution of a dietary supplement without the addition of standard substances and standard solutions of vitamins D_3_ and K_2_ were placed on the plate. The results obtained are presented in Table 2.

To sum up, under the established conditions, the concentration range for individual substances is from 0.4 to 6.0 µg/spot for vitamin D_3_, from 1.5 to 5.6 µg/spot for vitamin D_2_ and from 0.6 to 2.4 µg/spot for vitamin K_2_. The values of the regression coefficients reach values close to unity, which indicates a good correlation of the results. It has been shown that the method is characterized by good precision (RSD < 1%), and the obtained values of vitamin D_3_ and K_2_ recovery are in the range of 97.64–100.22%, which confirms the appropriate accuracy of the method.

**Figure 6:**
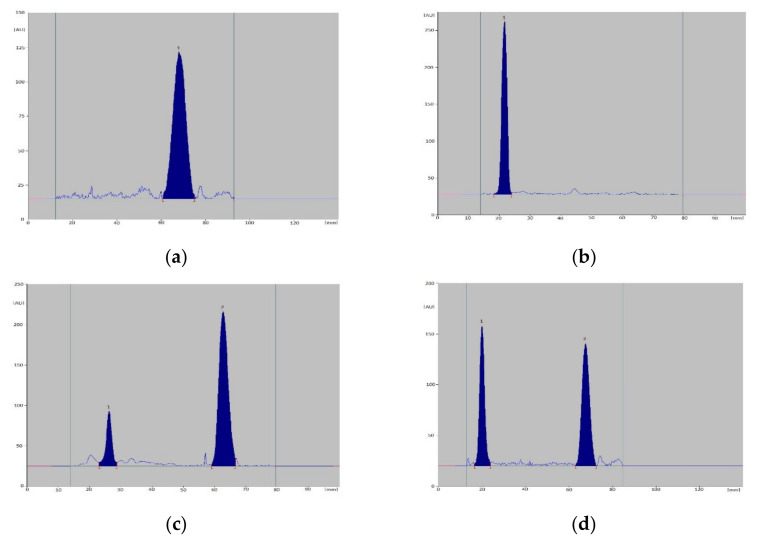
An example of sample densitograms registered for dietary supplements: Product A ((**a**); 1—D_3_), Product C ((**b**); 1—K_2_), Product F ((**c**); 1—K_2_, 2—D_3_) and Product K ((**d**); 1—K_2_, 2—D_3_).

Using the developed conditions, a quantitative analysis of selected dietary supplements containing vitamins D_3_ and K_2_ was carried out. The obtained results were analyzed using the Statistica (v.13.3, TIBCO Software Inc.), and the obtained results are summarized in Table 3. Sample densitograms recorded for the tested preparations are shown in Figure 6.

When analyzing the results, we wondered if selected dietary supplements that are advertised and sold to support or strengthen the immune system are accurately labeled and contain the content of active ingredients listed on the labels.

During the analysis of all preparations and standard solutions containing vitamin K_2_, stored for more than 48 h, an additional spot appeared on the chromatograms. This additional peak, with the retention factor R_F_ = 0.76, probably belonged to the decomposition product of vitamin. Its amount increased with the use of solutions stored for several days, even those stored in the refrigerator. This proves the relatively rapid decomposition of vitamin K_2_ in solution. Therefore, quantification of this compound was always carried out using freshly prepared solutions.

**Figure 7:**
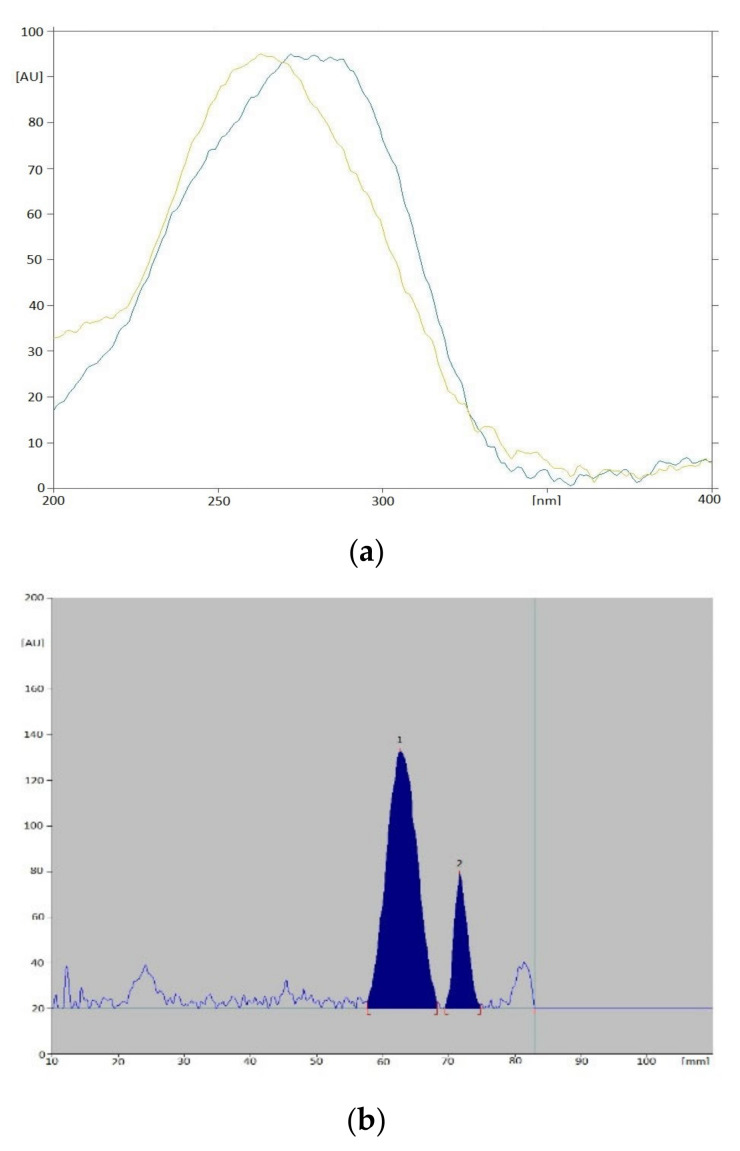
Absorption spectra in the UV range ((**a**); light green line—D_3_, dark green line—X) and an example densitogram ((**b**); 1—D_3_, 2—X) recorded for Product B.

Under analysis of Product B (in the form of a powder for solution) with more than 98% vitamin D_3_ content, additional spots were observed in the chromatograms. According to the manufacturer’s declaration, only vitamin D_3_ and calcium citrate should be present in this product. The densitogram and UV absorption spectra recorded for this product, presented on Figure 7, may suggest the presence of another component, named X. A similarity of the spectrum of the unknown component to the spectrum corresponding to vitamin D_2_ and similar R_F_ values can be here observed. It is only a suggestion, because unambiguous confirmation of its identity requires additional structural analyses.

During the analysis of all other dietary supplements (except Product B), no additional peaks were observed, apart from peaks from the determined vitamins D_3_ and K_2_. All supplements apart from Products A, P and S were within the expiration date when conducting the experiment. The reduced content of vitamins in these products (up to about 40% in Product S) may therefore be caused by their too long storage. The analysis of other supplements containing vitamin K_2_ showed a very diverse content in the analyzed supplements. In combination preparations containing both vitamins (D_3_ and K_2_), the determined amount of vitamin K_2_ turned out to be more satisfactory (above 87%). However, in supplements containing only vitamin K_2_, its content was much lower (approx. 44%), and in the case of Product D, the presence of this substance was not found. Therefore, it was decided to perform an additional test, increasing the concentration of the solution of this preparation by 5 times. Unfortunately, in this case, the presence of vitamin K_2_ was also not found, which may indicate its complete lack in the product or its minimal content, at the detection limit of the method. However, the content of vitamin D_3_ in the tested dietary supplements remained at a more satisfactory level. The smallest determined amount was about 62% of the content declared by the manufacturer (in the Product Z), while in other supplements, the content of vitamin D_3_ ranged from 71 to 101% (average 92%).

The carried-out analysis suggests that quality control measures were not sufficient for the majority of advertised dietary supplements containing vitamins D and K. For the majority of tested products, the claims made on the labels may be misleading to consumers, and the hypothetical performance of the purchased product will be significantly limited if it does not contain the ingredient (or contains too little of it) we expect. In this aspect, it can be concluded that there is a need for further and more extensive research that will allow to assess the real quality of available dietary supplements. New analytical procedures developed for this purpose will create new opportunities for comprehensive quality control analysis of these products. Especially if they present quick, simple and relatively cheap solutions and take into account the aspect of ‘green chemistry’, i.e., focusing on the design of processes that takes into account the reduction of analysis time and minimizes the use and generation of hazardous reagents.

## 4. Conclusions

The conditions for the separation of vitamins D_2_, D_3_ and K_2_ by thin-layer chromatography with the use of densitometric detection were optimized. As a result, a methodology was developed that allows for the determination of all three vitamins side by side. Then, the developed method was validated in accordance with the standards, stating that the method meets the requirements set for it and is characterized by appropriate specificity, sensitivity and reliability. Next, the established procedure was used for qualitative and quantitative analysis of dietary supplements in the form of tablets, powder, capsules and drops containing vitamins D_3_ and K_2_. The obtained scores as a result of the quantitative analysis show significant deviations in the content of vitamin K_2_ and D_3_ in the tested supplements. Of the 25 analyzed products, for 11, the content of the determined vitamins was above 95%. The results of the determinations and the verification of the obtained values with the quantity declared by the producers make us reflect on the quality of the products covered by the study. It also makes it possible to address the problem of the safety of using dietary supplements. Therefore, further research is recommended, including the reproducibility of samples from different production batches.

## Figures and Tables

**Table 1:** Method validation parameters with statistical evaluation specified for vitamin D_2_, D_3_ and K_2_.

Parameter		D_2_	D_3_	K_2_
regression equation		P = 3073.30 ∙ c + 29.88	P = 3399.20 ∙ c − 524.30	P = 5006.90 ∙ c + 521.17
r		0.9953	0.9997	0.9960
S_a_		134.38	38.78	225.10
S_b_		471.68	131.25	298.72
S_e_		630.80	217.89	425.20
residual analysis: regression equation		RR = −0.10 × 10^−3^ c + 0.98 × 10^−4^	RR = −0.10 × 10^−3^ ∙ c + 0.34 × 10^−3^	RR = 0.60 × 10^−4^ ∙ c − 0.50 × 10^−4^
residual analysis: r		−0.30 × 10^−6^	−0.20 × 10^−5^	0.10 × 10^−6^
Cook’s distance (av)		0.3410	0.2873	0.6215
Mahalanobis distance (av)		0.8571	0.8570	0.8333
LOD [μg/spot]		0.4604	0.1424	0.1790
LOQ [μg/spot]		1.5348	0.4748	0.5966
Direct precision (*n* = 9)	x_m_	4711.99	4914.45	3552.23
	SD	34.01	45.21	31.03
	RSD	0.72	0.92	0.87
Intermediate precision (*n* = 9)	x_m_	4462.33	4655.32	4097.07
	SD	30.00	43.41	35.94
	RSD	0.67	0.93	0.88

P—peak area; c—concentration [μg/spot]; r—correlation coefficient; S_a_—standard error of the slope; S_b_—standard error of the intercept; S_e_—standard error of estimation; RR—raw residuals; av—average value; x_m_—arithmetic mean; SD—standard deviation; RSD—relative standard deviation [%].

**Table 2:** Recovery values determined for vitamins D_3_ and K_2_ with statistical evaluation.

Level	D_3_	K_2_
80%	x_m_ = 99.03 SD = 0.1622, SDx_m_ = 0.0811 RSD% = 0.16	x_m_ = 98.95 SD = 0.5365, SDx_m_ = 0.2683 RSD% = 0.54
100%	x_m_ = 99.35 SD = 0.6312, SDx_m_ = 0.3156 RSD% = 0.64	x_m_ = 100.22 SD = 0.5819, SDx_m_ = 0.2910 RSD% = 0.58
120%	x_m_ = 97.64 SD = 0.4598, SDx_m_ = 0.2299 RSD% = 0.47	x_m_ = 98.24 SD = 0.6809, SDx_m_ = 0.3405 RSD% = 0.69

x_m_—arithmetic mean (*n* = 4); SD—standard deviation; SDx_m_—standard deviation of the arithmetic mean; RSD—relative standard deviation [%].

**Table 3:** The results of the analysis of the content of vitamins D_3_ and K_2_ in the analyzed dietary supplements along with the statistical evaluation.

Dietary Supplement	Determined Compound Declared Content	Determined Content [μg/1 tab., 1 dose, 1 caps. or 1 mL]	Statistical Evaluation (*n* = 8)
Product A	D_3_ 50 μg/1 tab.	x_m_ = 49.10	SD = 1.12, RSD = 2.28
Product B	D_3_ 5 μg/1 dose	x_m_ = 4.92	SD = 0.11, RSD = 2.24
Product C	K_2_ 100 μg/1 Table	x_m_ = 89.44	SD = 1.55, RSD = 1.73
Product E	K_2_ 100 μg/1 tab.	x_m_ = 92.25	SD = 1.52, RSD = 1.65
	D_3_ 50 μg/1 tab.	x_m_ = 49.11	SD = 1.11, RSD = 2.26
Product F	K_2_ 100 μg/1 tab.	x_m_ = 98.04	SD = 1.98, RSD = 2.02
	D_3_ 50 μg/1 tab.	x_m_ = 43.41	SD = 1.15, RSD = 2.64
Product G	D_3_ 100 μg/1 tab.	x_m_ = 95.66	SD = 0.83, RSD = 2.45
	K_2_ 75 μg/1 tab.	x_m_ = 73.95	SD = 1.85, RSD = 2.50
Product H	D_3_ 50 μg/1 tab.	x_m_ = 46.19	SD = 1.16, RSD = 2.52
	K_2_ 200 μg/1 tab.	x_m_ = 175.25	SD = 3.46, RSD= 1.97
Product I	D_3_ 50 μg/1 tab.	x_m_ = 42.78	SD = 0.99, RSD = 2.31
	K_2_ 75 μg/1 tab.	x_m_ = 68.70	SD = 1.40, RSD = 2.04
Product J	D_3_ 50 μg/1 tab.	x_m_ = 42.47	SD = 0.75, RSD = 1.77
	K_2_ 75 μg/1 tab.	x_m_ = 70.24	SD = 1.23, RSD = 1.75
Product K	D_3_ 50 μg/1 tab.	x_m_ = 45.28	SD = 0.92, RSD = 2.03
	K_2_ 75 μg/1 tab.	x_m_ = 69.85	SD = 1.10, RSD = 1.57
Product L	D_3_ 50 μg/1 tab.	x_m_ = 50.26	SD = 1.22, RSD = 2.43
	K_2_ 100 μg/1 tab.	x_m_ = 97.81	SD = 1.40, RSD = 1.43
Product M	D_3_ 50 μg/1 tab.	x_m_ = 46.92	SD = 0.84, RSD = 1.79
	K_2_ 100 μg/1 tab.	x_m_ = 92.66	SD = 1.02, RSD = 1.11
Product N	D_3_ 50 μg/1 tab.	x_m_ = 49.16	SD = 1.24, RSD = 2.52
	K_2_ 75 μg/1 tab.	x_m_ = 73.13	SD = 1.18, RSD = 1.61
Product O	D_3_ 50 μg/1 tab.	x_m_ = 48.54	SD = 1.19, RSD = 2.45
	K_2_ 75 μg/1 tab.	x_m_ = 74.07	SD = 1.57, RSD = 2.11
Product P	D_3_ 50 μg/1 tab.	x_m_ = 42.24	SD = 1.12, RSD = 2.65
	K_2_ 75 μg/1 tab.	x_m_ = 66.20	SD = 1.33, RSD = 2.00
Product Q	D_3_ 500 μg/1 mL drops	x_m_ = 456.67	SD = 1.97, RSD = 0.43
Product R	D_3_ 500 μg/1 mL drops	x_m_ = 358.07	SD = 1.48, RSD = 0.41
Product S	D_3_ 100 μg/1 caps.	x_m_ = 39.60	SD = 1.08, RSD = 2.74
Product T	D_3_ 80 μg/1 mL aerosol	x_m_ = 79.38	SD = 0.66, RSD = 0.83
Product U	D_3_ 25 μg/1 caps.	x_m_ = 24.85	SD = 0.14, RSD = 0.58
Product V	D_3_ 100 μg/1 caps.	x_m_ = 76.86	SD = 1.32, RSD = 1.72
Product W	D_3_ 25 μg/1 caps.	x_m_ = 20.55	SD = 0.35, RSD = 1.69
Product Y	D_3_ 100 μg/1 tab.	x_m_ = 101.88	SD = 1.53, RSD = 1.50
Product Z	D_3_ 50 μg/1 caps.	x_m_ = 31.16	SD = 0.62, RSD = 1.99

Table—tablet; caps.—capsule; x_m_—arithmetic mean; SD—standard deviation; RSD—relative standard deviation [%].

## Data Availability

Not applicable.

## Supplementary Materials

The following supporting information can be downloaded at: https://www.mdpi.com/article/10.3390/nu15071650/s1, Table S1: Optimization of the conditions for the separation of vitamins D_2_, D_3_ and K_2_.
