# Supplementary material for: Quality Control of the Dietary Supplements Containing Selected Fat-Soluble Vitamins D and K
Source: Nutrients. 2023 Mar 28;15(7):1650. doi: 10.3390/nu15071650 (PMC10096846; doi:10.3390/nu15071650)
Supplement: Supplementary file 1 [file nutrients-15-01650-s001.zip › nutrients-2295393-supplementary.pdf]

Table S1. Optimization of the conditions for the separation of vitamins D_2, D_3 and K_2.

Stationary phase	Mobile phase	Developing distance	Observations
TLC Silicagel 60F_{254}	n-hexane - ethyl acetate (9:1, v/v)	10 cm<br>15 cm	no separation
TLC Silicagel 60F_{254} activated with methanol or chloroform	n-hexane - ethyl acetate (9:1, v/v)	10 cm	compounds stay on the starting line
DC-Silicagel 60 RP-18 F_{254S};<br>DC-Alufolien Cellulose F	1% β-cyclodextrin - methanol (15:1, v/v)	10 cm	compounds stay on the starting line
DC-Silicagel 60 RP-18 F_{254S} activated at 60°C for 2h;<br>DC-Alufolien Cellulose F activated at 60°C for 24h	1% β-cyclodextrin - methanol (15:1, v/v)	10 cm	compounds stay on the starting line
DC-Silicagel 60 RP-18 F_{254S}	n-hexane - ethyl acetate (9:1, v/v)	10 cm	no separation D_2 and D_3
DC-Alufolien Cellulose F activated with mixture: 1% β-cyclodextrin- methanol (15:1)	n-hexane - ethyl acetate (9:1, v/v)	10 cm	moving with the eluent
DC-Silicagel 60 RP-18 F_{254S};<br>DC-Alufolien Cellulose F;<br>TLC Silicagel 60 F_{254}	1% β-cyclodextrin - methanol (15:1, v/v)	10 cm	compounds stay on the starting line
DC-Silicagel 60 RP-18 F_{254S};<br>DC-Alufolien Cellulose F;<br>TLC Silicagel 60 F_{254}	n-hexane - ethyl acetate (9:1, v/v)	10 cm	no separation
DC-Alufolien Kieselgur F_{254};<br>DC-Alufolien Polyamid 11 F_{254};<br>DC-Alufolien Aluminiunoxid 60 F_{254} neutral Typ E	toluene - acetone (1:10, v/v)	10 cm	moving with the eluent
DC-Alufolien Kieselgur F_{254};<br>DC-Alufolien Polyamid 11 F_{254};<br>DC-Alufolien Aluminiunoxid 60 F_{254} neutral Typ E	n-hexane - ethyl acetate (9:1, v/v)	10 cm	no separation D_2 and D_3
TLC Silicagel 60 F_{254} activated with 10% (v/v) paraffin in cyclohexane, dried 10 min at 60°C	methanol - water (19:1, v/v)	10 cm	D_2: R_F 0.63<br>D_3: R_F 0.61<br>K_2: R_F 0.24<br>D_2 + β-CD: R_F 0.63<br>D_3 + β-CD: R_F 0.59
TLC Silicagel 60 F_{254} activated with 10% (v/v) paraffin in cyclohexane, dried 10 min at 60°C	n-hexane - ethyl acetate (9:1, v/v)	10 cm	no separation D_2 and D_3
TLC Silicagel 60 F_{254} activated with 10% (v/v) paraffin in cyclohexane, dried 10 min at 60°C	1% β-cyclodextrin - methanol (15:1, v/v)	10 cm	compounds stay on the starting line
TLC Silicagel 60 F_{254} activated with 10% (v/v) paraffin in cyclohexane, dried 10 min at 60°C	methanol - water (17:3, v/v);<br>metanol	10 cm	slight differences in R_F for D_2 and D_3
TLC Silicagel 60 F_{254} activated with 10% (v/v) paraffin in cyclohexane, dried 10 min at 60°C	methanol - 1% β-cyclodextrin (19:1, v/v)	12 cm	no separation D_2 and D_3
TLC Silicagel 60 F_{254} activated with 10% (v/v) paraffin in cyclohexane, dried 10 min at 60°C	acetone – glacial acetic acid (3:2, v/v)	12 cm	no separation D_2 and D_3
TLC Silicagel 60 F_{254S}	acetone – glacial acetic acid (3:2, v/v)	12 cm	moving with the eluent
TLC Silicagel 60 F_{254} activated with 10% (v/v) paraffin in cyclohexane, dried 10 min at 60°C	methanol - water (19:1, v/v)	12, 15, 16, 20 cm	greater differences in R_F for D_2 and D_3 than over 10 cm path
TLC Silicagel 60 F_{254} activated with 10% (v/v) paraffin in cyclohexane, dried 10 min at 60°C;<br>TLC Silicagel 60 F_{254};<br>DC-Silicagel 60 RP-18 F_{254}	methanol - water (19:1, v/v)	12 cm	no separation D_2 and D_3

TLC Silicagel 60 F_{254} activated with 10% (v/v) paraffin in cyclohexane, dried 10 min at 60°C; TLC Silicagel 60 F_{254}; DC-Silicagel 60 RP-18 F_{254}	acetonitrile - methanol (7:3, v/v)	12 cm	no separation D_2 and D_3
TLC Silicagel 60 F_{254} activated with 10% (v/v) paraffin in cyclohexane, dried 10 min at 60°C	methanol - water (19:1, v/v)	12 cm	D_2: R_F 0.58<br>D_3: R_F 0.53<br>K_2: R_F 0.18
